# Writing in the air: A visualization tool for written languages

**DOI:** 10.1371/journal.pone.0178735

**Published:** 2017-06-02

**Authors:** Yoshihiro Itaguchi, Chiharu Yamada, Masahiro Yoshihara, Kazuyoshi Fukuzawa

**Affiliations:** 1 Department of System Design Engineering, Keio University, Yokohama, Japan; 2 Japan Society for the Promotion of Science, Tokyo, Japan; 3 Psychology Section, Faculty of Letters, Arts and Sciences, Waseda University, Tokyo, Japan; Arizona State University, UNITED STATES

## Abstract

The present study investigated interactions between cognitive processes and finger actions called “kusho,” meaning “air-writing” in Japanese. Kanji-culture individuals often employ kusho behavior in which they move their fingers as a substitute for a pen to write mostly done when they are trying to recall the shape of a Kanji character or the spelling of an English word. To further examine the visualization role of kusho behavior on cognitive processing, we conducted a Kanji construction task in which a stimulus (i.e., sub-parts to be constructed) was simultaneously presented. In addition, we conducted a Kanji vocabulary test to reveal the relation between the kusho benefit and vocabulary size. The experiment provided two sets of novel findings. First, executing kusho behavior improved task performance (correct responses) as long as the participants watched their finger movements while solving the task. This result supports the idea that visual feedback of kusho behavior helps cognitive processing for the task. Second, task performance was positively correlated with the vocabulary score when stimuli were presented for a relatively long time, whereas the kusho benefits and vocabulary score were not correlated regardless of stimulus-presentation time. These results imply that a longer stimulus-presentation could allow participants to utilize their lexical resources for solving the task. The current findings together support the visualization role of kusho behavior, adding experimental evidence supporting the view that there are interactions between cognition and motor behavior.

## Introduction

This study investigated interactions between cognitive processes and finger actions called “kusho,” which means “air-writing” in Japanese. Kusho is an action in which one moves his or her finger as a substitute for a pen to write with [1–5]. This can be regarded as a body-part-as-object (BPO) movement [6, 7]. Kusho behavior is elicited either consciously or unconsciously when individuals recall or memorize written languages such as the shapes of Kanji (Chinese) characters [1–3, 8, 9]. Kanji is a logographic symbol representing a lexical morpheme originally created in China and adopted into the Japanese language system. Sasaki [2] first reviewed the functional role of kusho behavior, which included his experimental studies published in Japanese. Until recently, no studies had been conducted on the functional role of kusho with behavioral data in controlled experiments. Brain activation patterns related to kusho behavior were discovered without a behavioral performance report [10, 11]. Recently, a behavioral experiment confirmed the facilitation effect of kusho behavior in learning situations; however, it was limited as it used a relatively small number of stimuli [3]. Furthermore, by using more controlled experimental settings and stimuli, it was revealed that watching the finger movements of kusho behavior may be important for its facilitative influence on the cognitive manipulation of written language [1]. Based on these studies, the present study further investigated the role of the visual feedback of finger movements on the kusho effect. In addition, we explored how the kusho effect is influenced by stimulus-presentation time and individual vocabulary size.

Kusho behavior is a sequential writing action, executed in the air or on a surface. Sometimes, when one employs kusho behavior, he or she watches the finger movement, but at other times, does not; i.e., the action is sometimes conducted completely out of sight, such as in the space at the lateral side of the body or on the knee. This behavior is frequently observed in individuals from a Kanji culture such as Chinese and Japanese [2, 4, 8, 12, 13]. In Japanese, most Kanji characters have more than one pronunciation and meaning, comprising several straight and curved lines (called strokes). Japanese children come to use kusho behavior when growing up and even individuals from non-Kanji cultures use kusho behavior if they have learned to write Kanji characters [2]. In addition, in experimental settings requiring the manipulation of shapes of Kanji characters and spelling of English words, almost all Japanese participants spontaneously used kusho behavior without having to be given any instructions about hand movements [1, 2]. Although the associated experimental data are unreported, it is not rare for participants to move their head or leg, instead of a finger, to do kusho when they are prohibited from conducting kusho behavior with their fingers. It has also been reported that second-language learners of Japanese use kusho behavior when they are trying to memorize complex and difficult Kanji characters [3, 14].

The clinical and educational benefits of kusho behavior are potentially high; the finger-writing action is likely to be an effective tool for brain-damaged patients and second-language learners. It has been revealed that alexia patients can read words and characters by tracing them with their finger, which they otherwise cannot read [15–18]. In contrast, it is also reported that agraphia patients cannot make use of kinetic movements to identify characters without seeing them [19–21]. Furthermore, Thomas [3] experimentally demonstrated that kusho behavior facilitated Kanji learning for second-language learners of Japanese. The clinical and educational benefits of finger actions may arise due to the common cognitive system for written language. Thus, to improve the efficiency of learning written language for children, second-language learners, and patients with language deficits, it is important to clarify how finger actions, including kusho behavior, influence the recognition of characters and words.

Although kusho behavior is very popular in daily life and has a potential impact on clinical and educational practice, little is known about how it interacts with cognitive processing. Sasaki [2] proposed that kusho has a functional role in assisting the visualization of characters or spelling (hereafter, referred to as the *visualization hypothesis*). Using a passive finger-tracing paradigm, where an experimenter guided a participant’s finger to trace a character or a figure, Yim-Yg et al. [13] provided partial support for the hypothesis. Using Sasaki’s [2] experimental paradigm, Itaguchi et al. [1] conducted a series of experiments in a more controlled setting. One of their experiments was a Kanji construction task with successive stimulus presentation. In the Kanji construction task, participants assembled a set of Kanji sub-parts to form an actual existing Kanji character [1, 2]; numerous Kanjis can be broken down into several sub-parts, with their own meanings and readings. This task requires visual manipulation of Kanji sub-parts in the mind. In their experiment, the Kanji sub-part stimuli were presented serially rather than simultaneously. The results demonstrated that watching kusho finger movements facilitated task performance, but kusho behavior executed out of participants’ sight did not. This result suggests that the visual feedback of kusho behavior is predominantly involved in task-solving, consistent with the visualization hypothesis. However, at the same time, they found that kusho behavior facilitated counting the number of strokes of a Kanji character, regardless of its visual feedback. These results suggest that the functional role of kusho behavior depends on the task, although the action itself is indistinguishable in different situations.

An additional purpose of this study was to investigate the relation between the visualization function of kusho behavior and cognitive factors, including memory demands in a task and individual vocabulary size. A previous study demonstrated that neither employing kusho behavior nor shortening the stimulus-presentation time affected the performance of the Kanji construction task when participants did not watch their finger movements [1]. This finding suggests that kusho behavior does not play a significant role in the memory retention of presented stimuli. It has been considered that kusho behavior might play a role in the memory retention process when one is involved in cognitive tasks [1, 2]. However, it has not been investigated how memory conditions affect task performance when participants watch their kusho behavior. In addition, although kusho behavior may provide positive effects for word processing for particular individuals, the influence of lexical properties such as vocabulary size on the facilitation effects of kusho behavior is completely unknown. It has been reported that children and Japanese learners from foreign countries exhibit kusho behavior as well as Japanese adults [2]. They can be regarded as having a small vocabulary. Furthermore, vocabulary size is assumed to be related to the proficiency of lexical processing [22]. On the basis of these reports, we assumed that the inefficient lexical system relies heavily on kusho behavior to solve difficult cognitive tasks related to written language [1]. However, this possibility has not been examined in the literature. Although the relevance to the kusho phenomenon was hypothetical, we considered it worth investigating, particularly in terms of its applicability for clinical and educational settings.

Analyzing task performance together with vocabulary size is helpful in understanding the requirements of a Kanji construction task and the facilitation effects of kusho behavior. At the neural level, greater activation was induced in a construction task than a copying task (simply transcribing a displayed character with an index finger) in the boundary area between the inferior parietal lobule and the occipital lobe, the premotor area, the pre-supplementary motor area, and the primary visual area [11]. This imaging study delineated a large visual demand in a construction task and kusho behavior also induced much activation of the brain areas responsible for visual processing. If kusho had an assistive role in visualization to form a character in the construction task, vocabulary size would be irrelevant to the kusho effect. On the contrary, it is possible that the facilitation kusho effects would be greater for individuals with a small vocabulary size compared with those with a large vocabulary size. Although the task requirements are different from the ordinary word-reading situation, it would be useful to investigate the relation between vocabulary size and the facilitation effects of kusho behavior to provide efficient programs for individuals with decreased lexical function.

To examine the relations between the visualization function of kusho behavior and memory demands as well as individual vocabulary size, we carried out a Kanji construction task [1, 2, 11] with a simultaneous stimulus-presentation. In this task, Kanji sub-parts were presented simultaneously for a specified time. After a presentation of the sub-parts, participants turned to view their right hand during a trial while thinking about their answer (eye-on-hand condition). Itaguchi et al. [1] also examined the kusho effect with simultaneous stimulus-presentation but only used an experimental condition in which participants did not watch their hand during a trial (eye-on-display condition), resulting in no significant effect of kusho behavior. On the other hand, with a successive stimulus-presentation paradigm, they showed a facilitation effect of kusho behavior by comparing two visual conditions, with and without the visual feedback of the hand. Thus, one may argue that the kusho effect depends on the stimulus-presentation method, rather than the presence of visual feedback. In the present study, to exclude this possibility, we applied a simultaneous presentation to the construction task with visual feedback of the hand. Employing the simultaneous presentation had several advantages. First, it contributes to confirming previous findings. Previous studies have observed kusho effects during the task with a successive presentation [1, 2], while the visualization hypothesis does not assume dependency on the stimulus-presentation method. Secondly, potential confounding factors arising due to presentation order can be eliminated. In the successive presentation, the order of presenting Kanji sub-parts can interact with the kusho effect; the successive presentation may cause priming effects as well as unbalance in memory decay among Kanji sub-parts [1]. In the task using simultaneous presentation, no such order effects would be expected.

Except for the experimental condition and analyses on the vocabulary size, experimental settings were the same as those used in Itaguchi et al. [1]. The number of correct responses (CR) was compared among two visual conditions (eye-on-hand and eye-on-display), three hand conditions (kusho, static, and circle drawing), and two stimulus-presentation time conditions (1 s and 3 s stimulus-presentations) [1]. The two time conditions were introduced to evaluate the effect of memory demands on the kusho effect. To assess individual vocabulary size, participants took a vocabulary test comprising a hundred Kanji words. This was done after they had completed the construction task.

## Method

### Participants

In total, 96 right-handed students from Waseda University participated in the experiment (20.1 years old, *SD* = 1.5). The average score on the Edinburgh handedness inventory was 89.3 (*SD* = 10.5). All participants were Japanese native speakers and had completed elementary and high school in Japan without any problems in reading and writing Japanese characters.

### Materials and procedure

#### Kanji construction task

**Fig 1:**
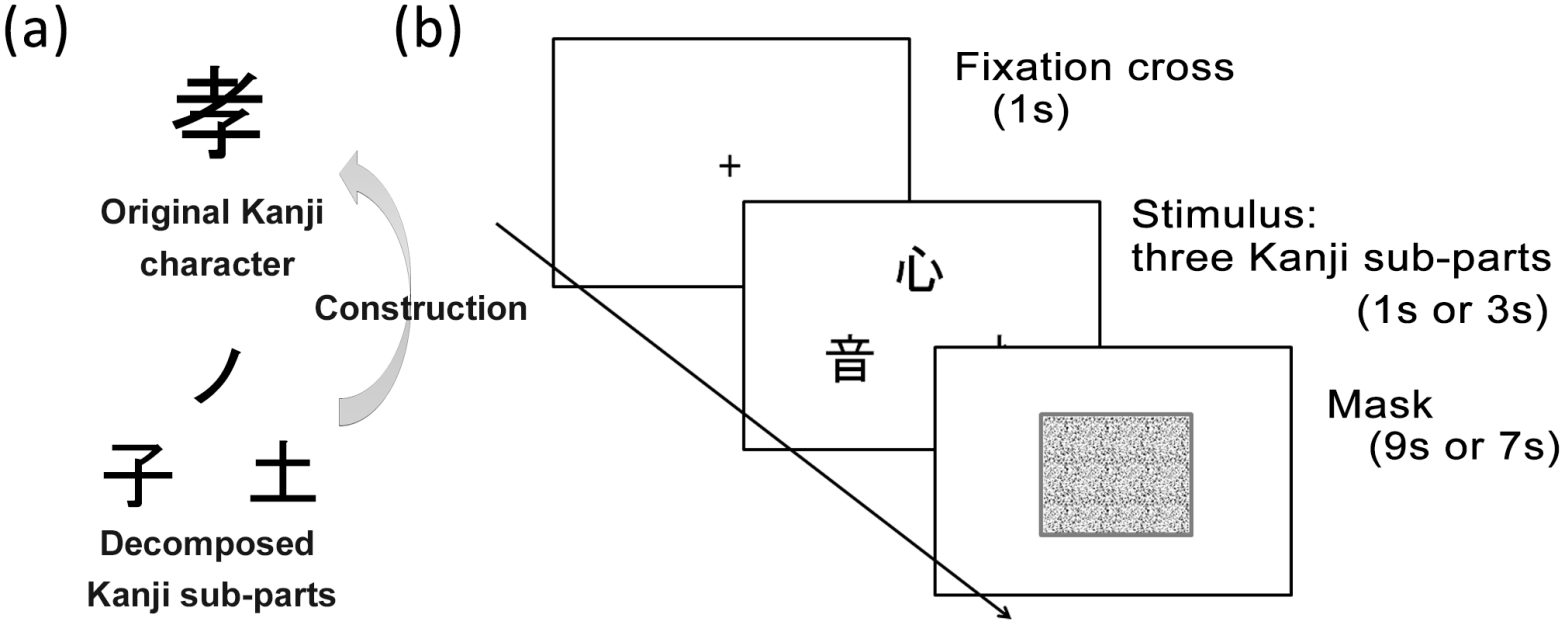
Experimental stimuli and sequence. (a) An original Kanji character and its sub-parts. In this study, one original Kanji character was always arranged into three sub-parts. In Fig 1a, for example, to make the original Kanji character, the three sub-parts had to be reduced in height (vertically compressed) while the top sub-part had to overlap with the other sub-parts. (b) The time course of one trial in the Kanji construction task.

Participants carried out a Kanji construction task wherein participants *construct* a Kanji character from several Kanji sub-parts presented like a puzzle [1, 2, 11]. In the present experiment, participants were asked to read aloud the original Kanji name as quickly as possible after three Kanji sub-parts were presented on a screen. Before the stimulus presentation, a fixation cross appeared for 1 s on the center of the screen. Simultaneously, the fixation cross vanished, three Kanji sub-parts, about 6 cm × 6 cm each in size, were presented on the center of the screen in a triangle formation (Fig 1a). After a specific amount of time had elapsed, the stimulus was erased and a mask image (of random dots) was shown (Fig 1b). From here, participants had to turn their eyes from the screen to their right hand (eye-on-hand condition) or to keep them on the screen (eye-on-display condition). This visual condition was a between-participant factor (*n* = 48 for each condition).

Participants were asked to read the original Kanji character into a microphone in front of them as quickly as possible. To form the Kanji character in their minds from the presented sub-pats, participants were allowed to overlap, expand, or reduce the presented sub-parts in size. In Fig 1a, for example, to make the original Kanji character, all sub-parts were reduced in height (vertically compressed) while the top sub-part was placed such that it overlapped with the other parts. Answering time was 10 s from the onset of stimulus presentation, regardless of the experimental conditions described below. We judged a response as correct when the response given by each participant was matched to the reading of the original Kanji and judged incorrect when it was wrong or not given in the answering time period.

To investigate the effect of kusho behavior on cognitive performance, we set three hand conditions (within participants). In the kusho condition, participants moved their right index finger freely in a manner resembling writing on the table. As an instruction, the participants were encouraged to employ kusho behavior to solve the task during the answering time. In the static condition, participants kept their right fist clenched on the table and did not move any of their fingers during the trial. In the circle-drawing condition, participants continued to make circular motions with their index finger during the trial. In this condition, a sheet of paper was placed under each participant’s right hand to show a circle that had to be traced. The radius of the circle was 4 cm, with a width of 1 cm. The left hand was always kept fisted on the table in all conditions. Two experimenters watched the behavior of the participants and the focus of their gaze through two webcams and a motion tracker system (Smarttrack, ART inc.). We assumed the static condition as a normal condition, which did not involve motor planning and visual and kinematic feedback. Further, the circle-drawing condition required motor planning and provided visual and kinematic feedback irrelevant to writing movements. This condition was introduced to eliminate the possibility that finger movements themselves would facilitate task performance.

In addition, to examine the effect of memory demands on task performance, we introduced two time conditions for the stimulus presentation (between participants, *n* = 48 for each condition). In one condition, a stimulus was presented for 1 s (1 s condition), and in the other condition the stimulus remained on display for 3 s (3 s condition). The participants were asked to watch the stimulus on the display until a mask appeared, and then to watch their right hand. We simply assumed that the memory demands were lower in the 1 s condition than in the 3 s condition, due to differences in stimulus-presentation time [1]. The 1 s condition was set because confirming three sub-parts requires at least 1 s [1, 23]. Presenting a stimulus for 3 s was assumed to enable participants to elaborate or activate more representations and still allowed them to watch the hand or finger movements during the rest of the answering time.

The participants answered 20 questions in each block under one of the hand conditions, and therefore, answered 60 questions in total (20 trials × 3 blocks). The order of hand conditions was counterbalanced among participants. Before starting the main trials, participants took several trials to become accustomed to the task and to be checked to ensure that they met the experimental requirements.

For the Kanji construction task, Kanji stimulus sets were created from that used in a previous study [1]. The previous study selected a total of 66 Kanji characters from the 1,006 Kanji characters which Japanese children learn in elementary school. The current experiment excluded six Kanji characters from the previous stimulus set due to the lower percentages of CR (less than 10%). Therefore, 60 Kanji stimuli were used in total. All the selected Kanji characters were broken down into three sub-parts (Fig 1a) and contained no parts that had the same reading as the original Kanji characters, to avoid possible phonetic facilitation due to similar readings between the original Kanji characters and their sub-parts [1, 2]. Only one original Kanji (i.e., one correct answer) was able to be constructed using the three sub-parts for each stimulus. The arrangement of sub-parts as a presented stimulus was changed from the relative relation in the original Kanji to prevent participants from coming up with the correct answer immediately (i.e., to control the difficulty of the task). For the same reason, the sub-parts in each stimulus were always presented in the same relative position. For three blocked hand conditions, three sets of Kanji characters (each containing 20 Kanji characters) were created to have the same character properties on average: familiarity [24], complexity [25], grade level learned in school, and difficulty [1]. Details of the stimulus sets are described in S1 Table. The three sets were randomly assigned to each hand condition and the order was counterbalanced.

#### Kanji vocabulary test

After completing the Kanji construction task, we assessed the participants’ Kanji vocabulary size using 100 RAKAN [26]. This test comprised a 100 Kanji word reading, which is standardized based on visual and phonetic aspects of word familiarity. The average score for Japanese university students was reported as 55.1 (*SD* = 14.8, *n* = 1407) [26]. In the present study, the average score of each participant group (*n* = 24 each) was 71.4 (*SD* = 6.1) and 68.5 (*SD* = 8.4) in the eye-on-hand condition and 68.6 (*SD* = 11.0) and 65.7 (*SD* = 10.9) in the eye-on-display condition. No significant difference was found in the scores among the four groups (*F* (3,92) = 1.42, *n*.*s*., *η*^2^ = 0.04) when a one-factor ANOVA was employed; this indicates that the participants in this study had almost the same vocabulary at the group level.

### Analysis

We calculated the number of CR in each condition. To exclude responses that were made 10 s after the stimulus presentation, we used recorded response time. Response time was defined as the time between the stimulus onset and the participant’s vocal onset, and was not analyzed because the stimulus-watching time differed between the two presentation time conditions. To examine the effects of hand condition and stimulus-presentation time, we carried out a three-factor mixed ANOVA (2 visual conditions × 3 hand conditions × 2 presentation time conditions) and multiple comparisons with Shaffer’s method as a post-hoc analysis [27]. In addition, we analyzed the interaction between task performance and individual vocabulary. First, to discuss the requirements of the Kanji construction task, we calculated the correlation coefficients between the number of CR in the construction task and the score of 100 RAKAN. Second, to examine the dependence of the facilitation effect of kusho behavior on vocabulary size, we calculated the correlation coefficients between the kusho effect and the 100 RAKAN score. The kusho effect was defined as the difference in CR between kusho and static conditions. To repeat correlation analyses, we applied Shaffer’s method to control type I error probability. We used R software (R Core Team, 2014) to perform the statistical analyses.

### Ethics statement

The study was conducted in accordance with the principles in the Declaration of Helsinki. The study and consent procedures were approved by the Ethics Committee on Human Research of Waseda University. All participants provided written informed consent.

## Results

Experimental results showed that CR in the kusho condition were produced to a greater degree over those in the static and circle-drawing conditions only in the eye-on-hand condition. It was also found that the number of CR in the static condition was greater than for that in the circle-drawing conditions. Neither the effect of stimulus-presentation time nor interaction between the hand condition and stimulus presentation time factors was observed in both visual conditions. Another analysis showed that the correlation between the number of CR and vocabulary score was moderately positive in the 3 s condition but not in the 1 s condition. Furthermore, the benefits of kusho behavior on the task performance and vocabulary score were found to be uncorrelated in all conditions.

**Fig 2:**
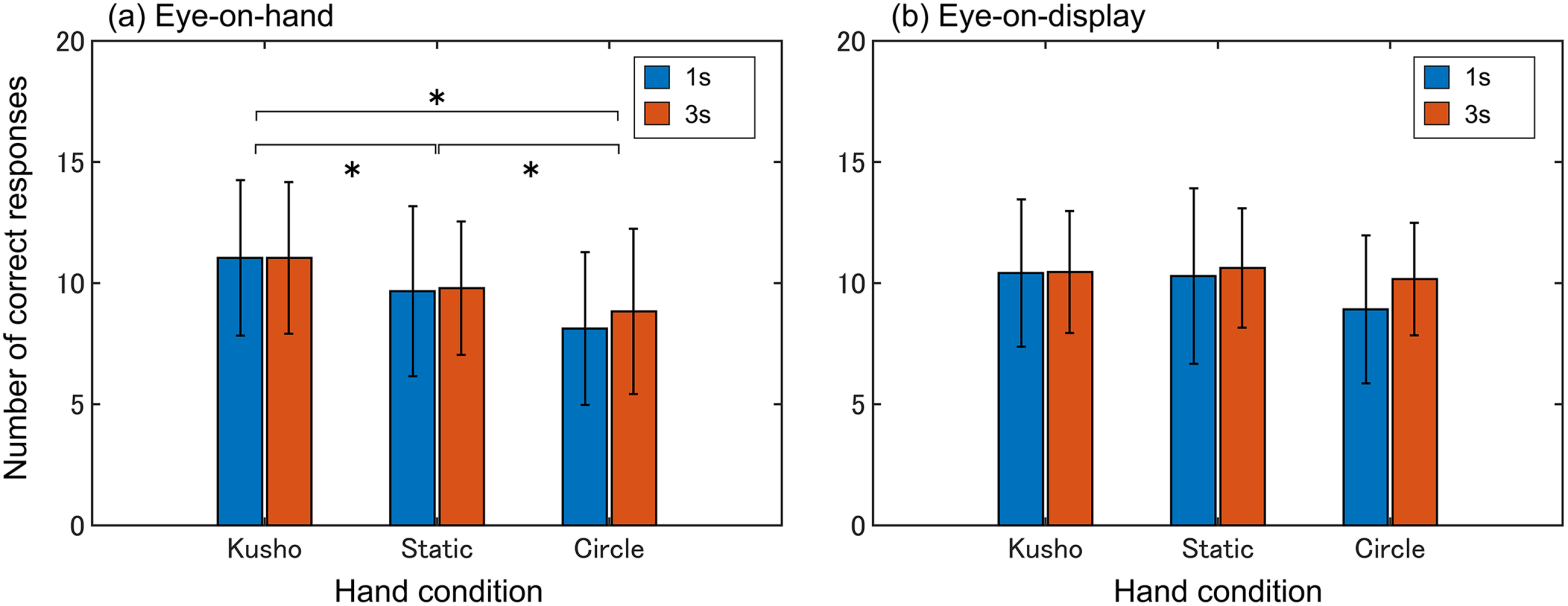
Average number of CR in the construction task. The simple main effect of the hand condition at the eye-on-hand condition was significant. The number of CR differed significantly among the three conditions only in the eye-on-hand condition.

First, we analyzed the number of CR. In Fig 2, the group averages and SDs of the number of CR are shown. The three-factor mixed ANOVA found a significant main effect for hand condition (*F* (2, 184) = 15.41, *p* < 0.001, *η*_p_^2^ = 0.14) and a significant interaction effect between visual and hand conditions (*F* (2, 184) = 3.92, *p* < 0.05, *η*_p_^2^ = 0.04). There were no significant effects for visual condition (*F* (1,92) = 0.57, *n*.*s*., *η*_p_^2^ = 0.01), presentation time (*F* (1, 92) = 0.62, *n*.*s*., *η*_p_^2^ = 0.01), the interaction effect between them (*F* (1, 92) = 0.06, *n*.*s*., *η*_p_^2^ = 0.00), the interaction effect between hand condition and presentation time (*F* (2, 184) = 1.28, *n*.*s*., *η*_p_^2^ = 0.01), and the three-factor interaction effect (*F* (2, 184) = 0.08, *n*.*s*., *η*_p_^2^ = 0.00). The simple main effect of the hand condition at the eye-on-hand condition was significant (*F* (2, 92) = 20.98, *p* < 0.001, *η*_p_^2^ = 0.31). The multiple comparisons revealed that the number of CRs in the kusho condition was greater than those of the static and circle conditions (*t* (46) = 3.30, *p* < 0.05, *d* = 0.48; *t* (46) = 6.23, *p* < 0.05, *d* = 0.90) in the eye-on-hand condition. Moreover, the number of CR in the static condition was significantly larger than that of the circle conditions (*t* (46) = 3.32, *p* < 0.05, *d* = 0.48).

Next, we analyzed the relation between the number of CR in the construction task and lexical test score. The correlation coefficients between the number of CR and the vocabulary score were summarized in Table 1. The correlation analysis showed that the number of CR of the construction task and vocabulary score had moderately positive correlations in the 3 s condition for the eye-on-hand condition. In contrast, the other coefficients were not significantly different from zero, except for the correlation coefficient in the kusho condition of the 3 s condition.

**Table 1:** The correlation coefficients between the vocabulary score and each performance score and their 95% confidential intervals. An asterisk (*) indicates a 5% statistical significance with Bonferroni’s correction.

	Eye-on-hand						Eye-on-display					
	1s			3s			1s			3s		
	*r*	95% CI		*r*	95% CI		*r*	95% CI		*r*	95% CI	
Kusho	.07	.03–.46	*n*.*s*.	.51	.13–.76	*	.13	-.30–.50	*n*.*s*.	.46	.14–.76	*
Static	.12	-.30–.50	*n*.*s*.	.50	.11–.75	*	.30	-.11–.62	*n*.*s*.	.29	-.08–.65	*n*.*s*.
Circle	.27	-.15–.60	*n*.*s*.	.70	.40–.86	*	.34	-.07–.66	*n*.*s*.	.36	-.06 –.66	*n*.*s*.
Kusho effect	-.07	-.48–.32	*n*.*s*.	.09	-.35–.45	*n*.*s*.	.18	-.24–.54	*n*.*s*.	-.14	-.51–.27	*n*.*s*.

Finally, to define the relation between the kusho effect and vocabulary score, we calculated the correlation coefficient between the difference of CRs (kusho minus static conditions) and the 100 RAKAN score. The correlation coefficients were indicated in Table 1. These correlation coefficients indicated that there were no particular relations between the kusho effect and vocabulary score. The scatter plots of the correlation analyses are available in the Supporting Information (S1 and S2 Figs).

## Discussion

The experiments provided two sets of novel findings. First, executing kusho behavior during the cognitive task improved performance regardless of memory demands as long as the participants watched their finger movements. This result supports the idea that visual feedback of kusho behavior helps in solving cognitive tasks. Second, at least in the condition where stimuli were presented for a relatively long time (3 s), performance in the Kanji construction task was positively correlated with vocabulary scores in the eye-on-hand condition. However, benefits from kusho behavior on the task performance and vocabulary test scores were uncorrelated. Moderate positive correlations between the task performance and vocabulary scores in the 3 s condition implied that a longer stimulus presentation could allow participants to utilize their lexical resources. In the following section, we first discuss the effect of kusho behavior on the performance of the Kanji construction task. We then discuss the relation between the task performance and vocabulary score. Finally, we discuss the relation between kusho effect and vocabulary score.

The present experiment showed that the execution of kusho behavior allowed participants to produce more correct answers in the construction task only when the visual feedback of finger movements was available. This result agrees with and also complements the previous work of Itaguchi et al. [1], which demonstrated the effect of visual feedback of kusho in the construction task with the successive stimulus-presentation method. It remained unclear whether there is an advantage of watching kusho behavior when the stimuli are presented simultaneously. The present study addressed this issue and successfully demonstrated the positive effect of watching kusho behavior on the task performance. Since the circle movement condition did not facilitate task performance, the increase in CR in the kusho condition cannot be attributed to a mere execution of finger movements. This demonstrates the importance of moving a finger in a writing manner in the cognitive processing of tasks. The present result is in line with previous findings that kusho behavior improved the performance in the construction task with successively presented stimuli only when participants observed finger movements during thinking [1]. These findings together suggest that not kinesthetic but visual feedback of kusho behavior helps in solving the construction task. This conclusion supports the visualization hypothesis for the functional role of kusho behavior [1, 2].

Kusho behavior affected the performance of visual manipulation of Kanji characters but the effect was somewhat limited. Cohen’s *d* for the kusho effect was 0.82 in the previous study using successive stimulus presentation [1] and 0.48 in the present study in the eye-on-hand condition using simultaneous stimulus presentation. The effect sizes were large and medium based on Cohen’s standard, respectively, indicating that kusho behavior provided a sufficiently positive influence on the task performance in terms of statistical standard. One might argue that the effect size was not overly large in terms of everyday sense for healthy individuals. In this experiment, the increase in CR due to kusho behavior was only 6.6% on average (1.3 out of 20 questions). The limited effect of kusho behavior in this study agrees with the facilitation effects of kusho behavior in learning situations [3]. Nevertheless, considering the high frequency of kusho occurrence, the impact of kusho behavior could be much larger. In experimental settings, it has been reported that almost all participants voluntarily and almost always unconsciously employ a kusho strategy while handling the Kanji construction task without any instructions about finger movements [1, 2]. This dissociation between the small effect size and frequent use of kusho behavior might be accounted for using the idea that finger movements lighten neural or cognitive load [1, 10, 28]. It should also be noted that the difference between studies in effect size (0.82 vs. 0.48) might have been caused by the experimental procedure: successive presentation vs. simultaneous presentation (see details in Itaguchi et al. [1]).

In the present study, there was no difference in the number of CR between the stimulus-presentation time conditions; this result does not contradict the visualization hypothesis. If kusho behavior has a functional role in maintaining the memory trace of presented stimuli, the kusho effect would be salient in the 1 s condition. In the current experiment, however, the degree of the kusho effect was almost the same regardless of the stimulus-presentation time. This result indicates that kusho behavior did not interact with memory retention processes, consistent with the previous study [1]. However, we cannot reject the possibility that kusho behavior plays an important role in memory retention. In the static condition, the number of CR did not differ by the stimulus-presentation time condition, which implies that the experimental setting did not sufficiently control memory requirements of the stimuli, at least on a behavioral level. Therefore, although it is likely that there were adequate differences in memory demands due to the experimental manipulation of stimulus-presentation time, we did not provide any behavioral evidence for it. Nevertheless, we observed positive kusho effects in both the stimulus-presentation time conditions and these effects did not interact with the presentation time condition. As the visualization processes of the presented stimulus were not assumed to interact with memory retention processes, the lack of the effects of stimulus-presentation time neither damage our findings nor the visualization hypothesis.

The strong coupling between symbol and motor actions may underlie the fact that kusho behavior often appears in everyday life and assists visual manipulation. The previous findings [1, 2] and the current results together suggest that the facilitation effect of kusho behavior might be caused by efficient visual manipulation due to obtaining external visual feedback. A visual feedback allows participants to confirm the results of mental manipulation of Kanji characters more easily; the visual image in mind can be explicitly confirmed in the external visual space, otherwise, it remains obscure. This may be in accord with another daily-life experience; we often actually type or “air-type” a series of keys sequentially before we retrieve an incredibly complex password. Common mechanisms might be involved in this typing behavior and kusho behavior. Previous studies have suggested that the repeated writing experience of learning situations forms a tight coupling between motor representations and character representations [2, 29, 30]. Moreover, it has been reported that typing is selectively impaired by brain damage [31–33], the cause of which could be attributed to dysfunction of the strong connection between orthography and motor representation. Siok et al. [34] suggested that the system of a written language constrains the connection between reading and writing. In line with the study, it has been shown that a neural overlap exists between reading and writing in Kanji characters [30] as well as reading and typing in English words [35]. These studies together suggest that our brain is tuned to depend on external feedback to at least recall complex visual symbols, which have been learned through executing physical actions. Therefore, such strong coupling between symbols and motor actions may contribute to the frequent use and the facilitation effect of kusho behavior.

Second, this study, for the first time, reports the relation between the performance of a Kanji construction task and vocabulary scores. Previous studies used the task but did not consider vocabulary size while discussing task performance [1, 2, 11]. We found that the relation between task performance and vocabulary size differed by stimulus-presentation time. When stimuli were presented for 3 s, the task performance was positively correlated with vocabulary scores regardless of the hand condition in the eye-on-hand condition. In contrast, in the 1 s conditions of both visual conditions, the task performance and vocabulary score were uncorrelated. Although the vocabulary score might only reflect lexical size and, thus, might not be the critical variable for the task, our results at least suggest that it is important to introduce vocabulary size as a variable to further understand the relation between kusho behavior and the construction task.

The discrepancy in the correlation coefficient between the stimulus-presentation time conditions might be interpreted in terms of lexical networks; the activation of the lexical network could achieve a stronger and more stable state by a longer stimulus observation. In the processing of an input stimulus, activation of the lexical network is automatically propagated in a bottom-up and interactive manner and settles into a certain stable state [36]. Lexical access to a single word was suggested to be completed before 500 ms from stimulus presentation [37–39] and the process would be sequential [40]. It was also reported that a brief presentation of a sub-part of a Kanji character (i.e., a prime) influenced lexical processing of the following stimulus that possessed the same sub-parts [41]. In the current paradigm, we can regard the presented Kanji sub-parts as primes for the original character, which work to activate the lexical network and aid the determination of the original character. In the 3 s condition, there would be sufficient time to activate the network from each of the three Kanji sub-parts, and thus, the network would be ready to provide appropriate candidates for the target character, whereas in the 1 s condition, sufficient time might not be available. It is also plausible to assume that the activation of the lexical network might be more extended for individuals with a high vocabulary score because they would be able to activate more words [22]. These assumptions may explain the discrepancy of correlation coefficients induced by the presentation time condition; in the 3 s condition, participants with a high vocabulary score would have benefited from their lexical ability in solving the task, whereas in the 1 s condition they would not have benefited because the network might have been still unstable. However, the present results are still difficult to interpret because the average number of CR did not differ between the 1 s and 3 s conditions as well as because there was no direct comparison of the correlation coefficients between 1 s and 3 s conditions. If a longer stimulus-presentation time makes the lexical network more efficient, CR would be produced more in the 3 s condition than in the 1 s condition. These explanations are parsimonious but not well grounded by experimental evidence, which need to be carefully examined.

Third, the benefits from kusho behavior on the task performance and vocabulary size were not significantly correlated and had a very small correlation coefficient (0.07 and −0.09 in in the eye-on-hand condition and 0.18 and −0.15 in the eye-on-display condition). If kusho exclusively contributed to the visualization of the shapes of Kanji characters in the construction task, vocabulary size would not be correlated with the facilitation effect of kusho behavior. Our experimental results are consistent with this inference. Sasaki [2] found that the kusho effect was larger on the table than in the air, implying the importance of the visibility of the trajectory of the movement. Itaguchi et al. [1] demonstrated that the kusho effect was evident only when participants watched their finger movements. Even using a different stimulus-presentation method from these previous studies, the present study showed that kusho behavior had facilitation effects on the task performance when participants watched their finger movements. It is also known that visually-related brain areas were strongly activated during the Kanji construction task [11]. The null result of the correlation coefficient between kusho effect and vocabulary size does not contradict these studies which proposed the idea that the functional role of kusho behavior is exclusively involved in visual manipulation processing in the Kanji construction task. That is, the primary role of the kusho behavior may be to visualize and then externally confirm the imagined symbol. However, the vocabulary score of the participants in the present experiment was much higher than the standard for Japanese students, which could contribute to the lack of a significant relation between the kusho benefits and vocabulary size. This sampling issue limits the generalizability of the findings in the present study to the individuals with relatively low vocabulary size.

This study, meanwhile, suggests that kusho behavior is a candidate tool for individuals with disorders or immaturity of visualization function in recalling written language, if they watch their finger actions. Although we did not demonstrate that the kusho effect is very huge in an everyday sense, it could have a sufficient impact in terms of cost performance, particularly for those who have a low level of function for visual manipulation. It has been known that alexia patients can recognize a character that is kinetically presented as a series of passive finger movements [20, 42]. This is interpreted as an example that kinetic information through movements elicits internal representations of written languages. Furthermore, it was reported that alexia patients read characters by observing the other person writing the character [43]. These neuropsychological symptoms suggest that creating a visual image of characters in the mind is important while reading or writing them [44]. This creation would be assisted by observing writing movements. As for learning situations, Thomas [3] reported a facilitation effect of kusho behavior for second-language learners. Although the participants’ gaze was not reported in that study, the visualization hypothesis suggests that the learning effect would have been greater if learners had watched their kusho movements more closely. To further understand the nature of kusho behavior, more studies are needed on the application of kusho behavior for beginners’ learning and patients’ re-learning a language.

The visualization role of kusho behavior may have theoretical significance in terms of the frameworks for BPO behavior [6, 7] and embodied cognition [45, 46]. Kusho is regarded as one of the most frequently seen BPO behaviors in daily life, since the finger movements of kusho behavior substitute for writing movements with a pen, despite the fact that it is not in the shape of a hand holding a pen. Such substitution suggests that there are motor representations for the point of action of habitual behaviors in our brain that are independent of body effectors. In the case of writing, the point of action is the pen’s tip. The present study confirmed that the facilitation effect of kusho appeared when the visual feedback of the behavior was available, whereas it did not appear when visual feedback was not allowed [1]. These findings suggest that the point of action for a BPO behavior is represented in the external coordinates, which brings positive influences on cognitive processing when appropriately visualized. The kusho effect observed in the present study also agrees with the framework of embodied cognition [45, 46], which argues that bodily action confines cognitive or perceptual processing [47–49]. In the present study, the task performance in the kusho condition was higher than that in the static condition, whereas the performance in the circle-drawing condition was lower than in the static condition. This result may support the idea of embodied cognition because the cognitive performance was found to depend on the type of bodily movement. However, the importance of visual feedback relating to movements in the kusho effect may not immediately fit with the idea of embodied cognition. Although the visual dependence of bodily actions for efficient cognitive processing sheds new light on each theoretical domain, these discussions are beyond the scope of the present study and require further extensive research.

In conclusion, this study further provided evidence for the visualization function of kusho behavior. Executing kusho behavior would improve cognitive task performance when participants watch the finger movements, regardless of their vocabulary size and the stimulus-presentation time. In addition, a longer stimulus presentation might allow participants to use lexical resources, suggesting that vocabulary size is one of the important task requirements and should be considered. However, benefits from kusho behavior and vocabulary size are likely not correlated, at least for participants with normal or relatively high lexical ability. These findings together agree with the idea that a visual feedback of kusho behavior helps to solve cognitive tasks. The findings imply that we better not miss our bodily movements when we perform them and that kusho behavior might be a useful phenomenon in understanding the interaction between the body and cognitive processes.

## Supporting information

S1 Fig Relation between the construction task performance and Kanji reading score. (TIF)

S2 Fig Relation between the kusho effect of the construction task performance and Kanji reading score. (TIF)

S1 Table Three sets of Kanji characters used in the experiment and average profiles of each set. The difficulty is based on the average number of correct responses in the static condition in a preliminary experiment conducted in our previous study (Itaguchi et al. 2015).
(DOC)
