# Supplementary figures and images for: Writing in the air: A visualization tool for written languages
Source: PLoS One. 2017 Jun 2;12(6):e0178735. doi: 10.1371/journal.pone.0178735 (PMC5456354; doi:10.1371/journal.pone.0178735)

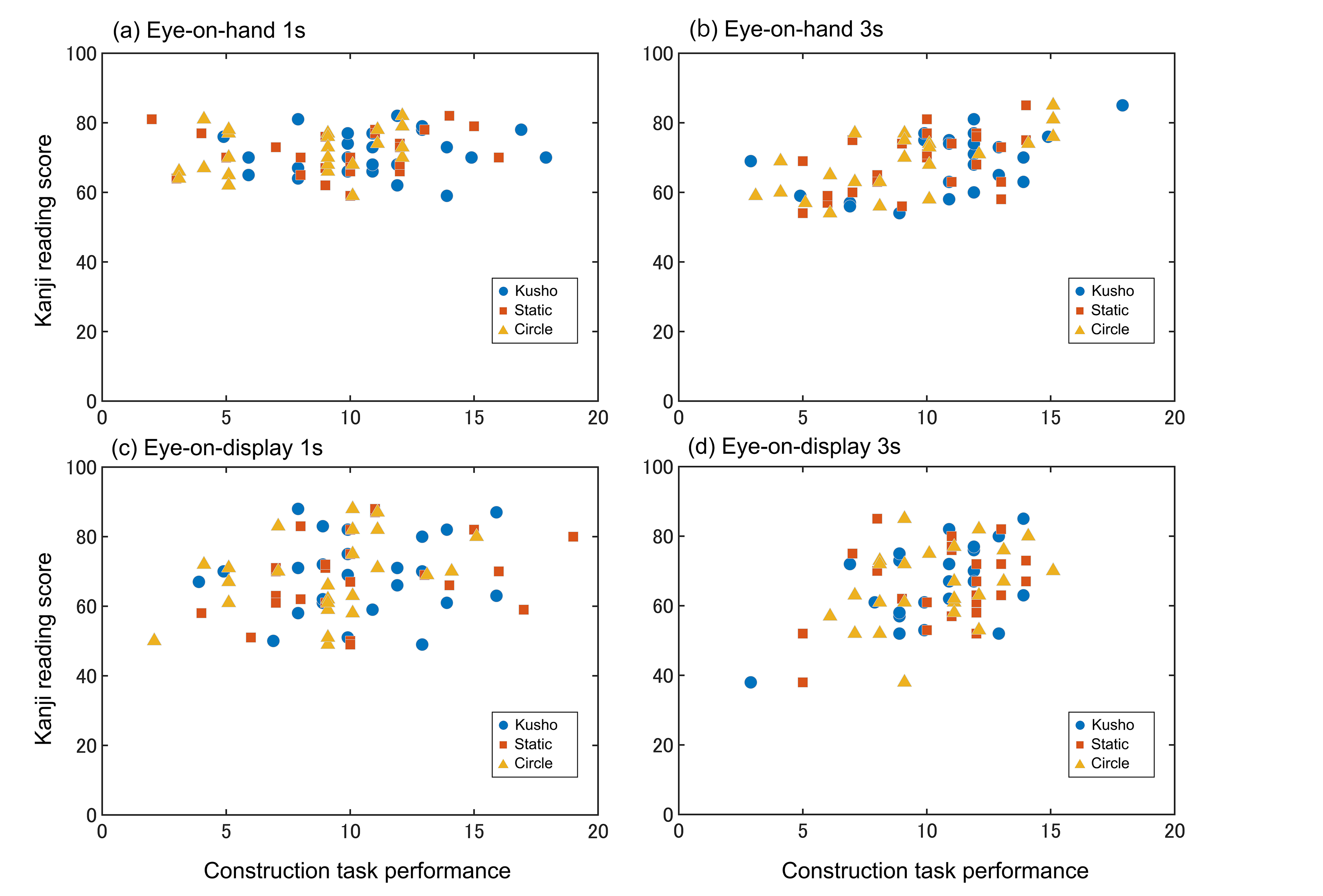

Supplement: S1 Fig — (TIF) [file pone.0178735.s001.tif]

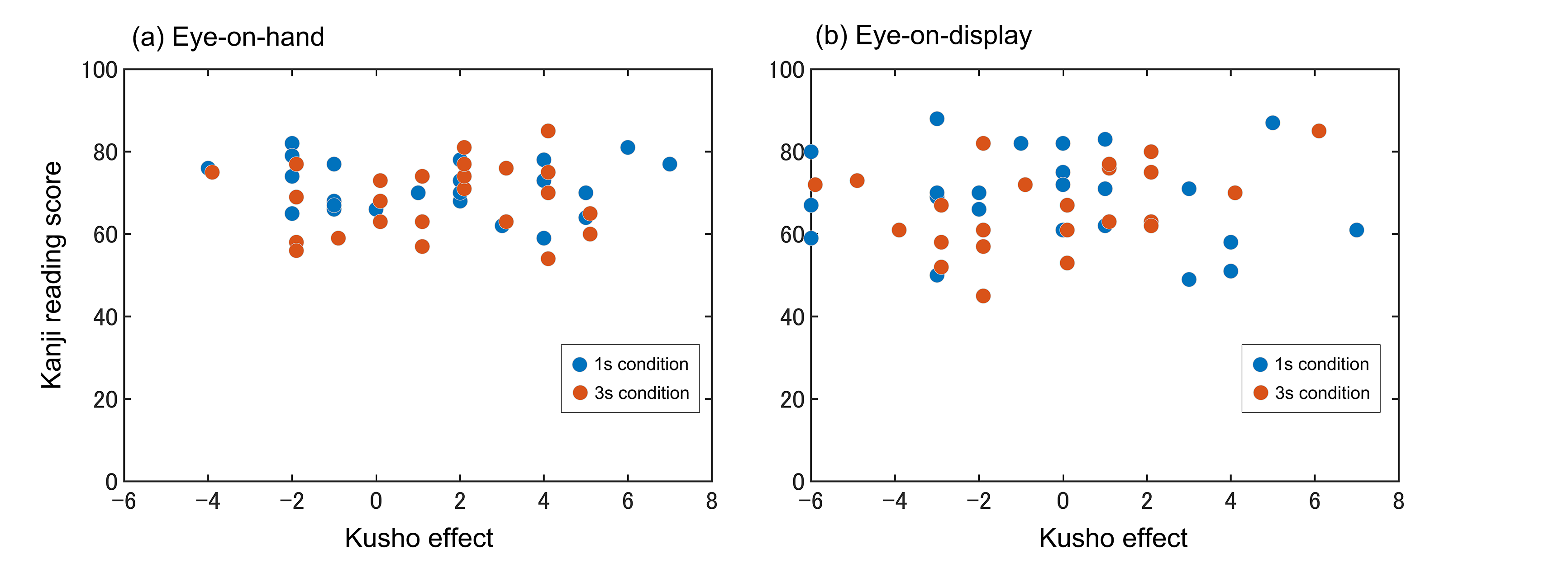

Supplement: S2 Fig — (TIF) [file pone.0178735.s002.tif]
