# Supplementary material for: Writing in the air: A visualization tool for written languages
Source: PLoS One. 2017 Jun 2;12(6):e0178735. doi: 10.1371/journal.pone.0178735 (PMC5456354; doi:10.1371/journal.pone.0178735)
Supplement: S1 Table — The difficulty is based on the average number of correct responses in the static condition in a preliminary experiment conducted in our previous study (Itaguchi et al. 2015). (DOC) [file pone.0178735.s003.doc]

**Supporting Information**

**S1 Table. Three sets of Kanji characters used in the experiment and average profiles of each set. The difficulty is based on the average number of correct response in the static condition in a preliminary experiment conducted in our previous study (Itaguchi et al. 2015).**

|  | **Set A** | **Set B** | **Set C** |
| --- | --- | --- | --- |
| 1 | 恩 | 害 | 親 |
| 2 | 型 | 賀 | 暗 |
| 3 | 路 | 詩 | 校 |
| 4 | 保 | 略 | 数 |
| 5 | 辞 | 敬 | 強 |
| 6 | 資 | 案 | 尊 |
| 7 | 勇 | 姿 | 格 |
| 8 | 導 | 線 | 孝 |
| 9 | 曜 | 接 | 特 |
| 10 | 量 | 唱 | 雑 |
| 11 | 染 | 章 | 綿 |
| 12 | 鼻 | 混 | 貯 |
| 13 | 孫 | 具 | 宿 |
| 14 | 昭 | 始 | 盟 |
| 15 | 界 | 想 | 筋 |
| 16 | 程 | 標 | 貴 |
| 17 | 操 | 認 | 賃 |
| 18 | 指 | 軽 | 聖 |
| 19 | 新 | 謝 | 葉 |
| 20 | 松 | 首 | 設 |
| Familiarity | 5.4±0.7 | 5.5±0.6 | 5.4±0.8 |
| Complexity | 4.6±0.7 | 4.5±0.7 | 4.6±0.5 |
| Grade in school | 4.1±1.2 | 4.0±1.2 | 4.4±1.6 |
| Difficulty | 0.6±0.2 | 0.6±0.2 | 0.6±0.2 |
